# Influence of Mechanical Unloading on Articular Chondrocyte Dedifferentiation

**DOI:** 10.3390/ijms19051289

**Published:** 2018-04-25

**Authors:** Simon L. Wuest, Martina Caliò, Timon Wernas, Samuel Tanner, Christina Giger-Lange, Fabienne Wyss, Fabian Ille, Benjamin Gantenbein, Marcel Egli

**Affiliations:** 1 Lucerne University of Applied Sciences and Arts, School of Engineering and Architecture, Institute of Medical Engineering, Space Biology Group, CH-6052 Hergiswil, Switzerland; simon.wueest@hslu.ch (S.L.W.); martina.calio@hslu.ch (M.C.); timon.wernas@hslu.ch (T.W.); samuel.tanner@hslu.ch (S.T.); christina.giger@hslu.ch (C.G.-L.); fabienne.wyss@hslu.ch (F.W.); fabian.ille@hslu.ch (F.I.); 2 University of Bern, Institute for Surgical Technology and Biomechanics, Tissue and Organ Mechanobiology, CH-3014 Bern, Switzerland; benjamin.gantenbein@istb.unibe.ch

**Keywords:** articular chondrocytes, bovine primary cells, dedifferentiation, mechanosensitive ion channel, qPCR, *TRPC1*, *TRPV4*, random positioning machine (RPM), simulated microgravity

## Abstract

Due to the limited self-repair capacity of articular cartilage, the surgical restoration of defective cartilage remains a major clinical challenge. The cell-based approach, which is known as autologous chondrocyte transplantation (ACT), has limited success, presumably because the chondrocytes acquire a fibroblast-like phenotype in monolayer culture. This unwanted dedifferentiation process is typically addressed by using three-dimensional scaffolds, pellet culture, and/or the application of exogenous factors. Alternative mechanical unloading approaches are suggested to be beneficial in preserving the chondrocyte phenotype. In this study, we examined if the random positioning machine (RPM) could be used to expand chondrocytes in vitro such that they maintain their phenotype. Bovine chondrocytes were exposed to (a) eight days in static monolayer culture; (b) two days in static monolayer culture, followed by six days of RPM exposure; and, (c) eight days of RPM exposure. Furthermore, the experiment was also conducted with the application of 20 mM gadolinium, which is a nonspecific ion-channel blocker. The results revealed that the chondrocyte phenotype is preserved when chondrocytes go into suspension and aggregate to cell clusters. Exposure to RPM rotation alone does not preserve the chondrocyte phenotype. Interestingly, the gene expression (mRNA) of the mechanosensitive ion channel *TRPV4* decreased with progressing dedifferentiation. In contrast, the gene expression (mRNA) of the mechanosensitive ion channel *TRPC1* was reduced around fivefold to 10-fold in all of the conditions. The application of gadolinium had only a minor influence on the results. This and previous studies suggest that the chondrocyte phenotype is preserved if cells maintain a round morphology and that the ion channel *TRPV4* could play a key role in the dedifferentiation process.

## 1. Introduction

Due to the limited self-repair capacity of articular cartilage, the surgical restoration of defective cartilage remains a major clinical challenge [1,2,3]. In the clinically used, cell-based approach, known as autologous chondrocyte transplantation (ACT), chondrocytes are extracted from healthy tissue biopsies and are expanded in vitro in monolayer culture. When reaching a sufficient number of cells, the chondrocytes are implanted back into the cartilage defect of the patient [4,5,6]. However, this approach has limited success [6,7,8,9,10,11], possibly because the chondrocytes acquire a fibroblast-like phenotype in monolayer culture [7,12,13,14,15,16,17,18,19]. This adaptation process, which is called dedifferentiation, is characterized by a decrease in collagen type II (*COL2*) and an increase in collagen type I (*COL1*) expression. Similarly, the proteoglycans aggrecan (*ACAN*) and versican (*VCAN*) are downregulated and upregulated with progressing dedifferentiation respectively [12,13,14,15,17,20]. Therefore, the ratio of *COL2*/*COL1* and *ACAN*/*VCAN* expression is typically used as a dedifferentiation maker [12,16,17,21]. In tissue engineering research, dedifferentiation is addressed by using three-dimensional scaffolds (such as agarose gel [22], alginate beads [15,23,24,25], collagen gel [26,27]), pellet culture [14,25,28], and/or the application of exogenous factors, such as members of the transforming growth factor (TGF family) [28,29,30].

Disuse or prolonged mechanical unloading of cartilage leads to accelerated degeneration [31,32,33]. For this reason, in vitro bioreactors have been developed to apply mechanical loading to tissue engineered samples [34,35]. Much less is known about mechanical unloading in low gravity. Because cartilage and chondrocytes are highly mechanosensitive [36], several experiments were performed under real and simulated microgravity (or weightlessness) to explore the effect of mechanical unloading. Simulated microgravity is typically generated by ground-based devices, which intend to mimic a weightless condition [37]. Tissue-engineered bovine cartilage that was cultivated for four months aboard the Russian Mir Space Station was more spherical, smaller, and mechanically inferior when compared with the ground control samples [38]. In a later experiment, porcine chondrocytes were cultured in microgravity on the International Space Station (ISS) and simulated microgravity using the random positioning machine (RPM), which is a ground-based microgravity simulation that will be introduced below [39]. The space-flown chondrocytes exhibited a reduced, discontinuous matrix with a reduced deposition of proteoglycans when compared with the ground control. Also, the cellular density was significantly decreased in space. However, the gene expression ratios of collagen type II/I were higher and the ratio of aggrecan/versican was lower in the space-flown samples than in the ground samples. The samples exposed to simulated microgravity on the RPM generally displayed intermediate effects [39].

In a ground-based RPM study, human chondrocytes revealed an increased amount of collagen type II protein expression compared with static controls [40]. In a similar experiment, chondrocytes showed decreased collagen type I and increased collagen type II and aggrecan protein synthesis [41]. Longer cultivation experiments on the RPM revealed that chondrocytes begin to form three-dimensional cell clusters [41]. This was also observed on other ground-based microgravity simulation devices, namely the fast-rotating clinostat and the rotating wall vessel (RWV) [42]. Both of the devices rotate the samples around a horizontal axis (in contrast to the RPM, which rotates the samples around two axes). Whereas, specific fast-rotating clinostat designs allows to rotate adherent cells, samples that were exposed to the RWV are typically seeded as suspended cells, and the rotation speed is adjusted such that the cells always remain in suspension [43,44]. Human articular chondrocytes that were cultured for 90 days in the RWV formed about 1 cm large three-dimensional assemblies. Apart from a fibrous outer layer, the assembly was stained positive for collagen type II and negative for collagen type I [45,46]. In conclusion, chondrocyte dedifferentiation “maker” *COL2*/*COL1* was higher under real and simulated microgravity compared with static ground controls. Therefore, real and simulated microgravity could possibly retard phenotype dedifferentiation.

The RPM is used (among other devices) as a ground-based model for long-term microgravity simulation [37,47,48,49]. Because multiple cell types, including chondrocytes, form three-dimensional cell aggregates on the RPM, the RPM is now expanding to tissue engineering applications as well [47,50]. The RPM consists of a gimbal-mounted platform onto which samples are mounted and are continuously rotated about two perpendicular axes. In this study, we examined if the RPM is an applicable treatment for expanding chondrocytes in vitro in such a way that they maintain their phenotype. Additionally, during the progress of dedifferentiation, transient receptor potential (TRP) channels are regulated in chondrocytes [51,52]. Because the TRP family has several mechanosensitive members [53], we also examined whether RPM exposure modulates the expression of two mechanosensitive channels, namely *TRPC1* (transient receptor potential channel 1) and *TRPV4* (transient receptor potential cation channel subfamily V member 4). Both channels are well expressed in chondrocytes [54,55]. *TRPV4* appears to play a central role in chondrogenesis, as *TRPV4* positively regulates the chondrogenic transcription factor *SOX9* [56]. Furthermore, we speculated that gadolinium (Gd^3+^), which is a nonspecific ion-channel blocker, could influence chondrocyte dedifferentiation.

## 2. Results

**Figure 1:**
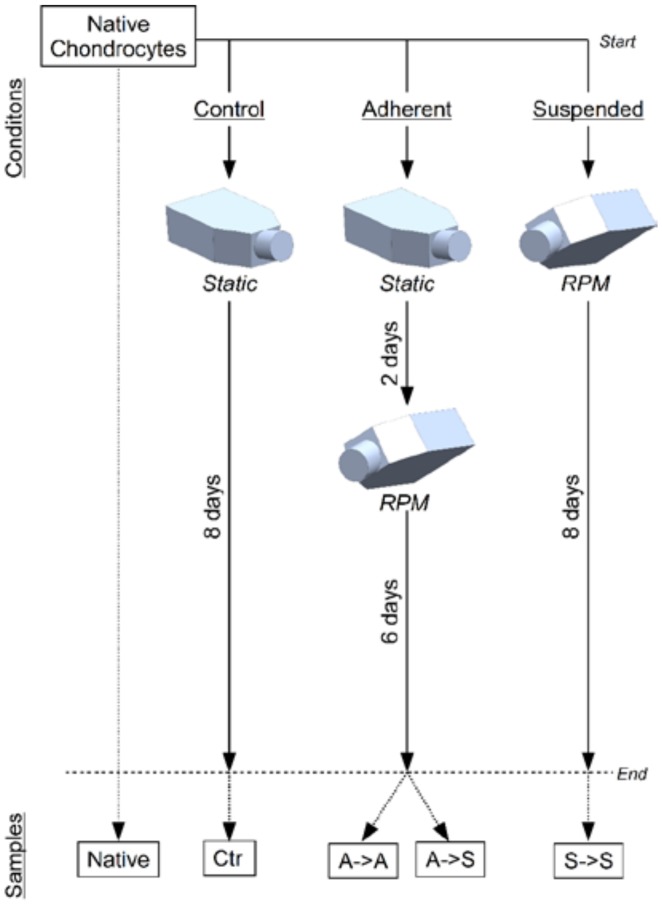
Experiment design. Bovine chondrocytes were distributed to six experimental groups in commercial T25-flasks. The control group was left for eight days in static monolayer culture (Ctr). The “adherent” groups were kept for two days in monolayer culture and subsequently exposed to the RPM for six days. The “suspended” groups were immediately placed on the RPM for eight days (S->S). After the experiment, the samples were collected for further analysis. For the “adherent” groups, the adherent cells (A->A) were collected separately from the cells that became suspended (A->S). In addition, freshly thawed cells were lysed for gene expression analysis, which was considered to represent the “native” chondrocytes.

Primary bovine chondrocytes were distributed to six experimental groups (Table 1 and Figure 1). Control groups were incubated for eight days in static monolayer culture (Ctr). “Suspended” groups were exposed for eight days to RPM rotation (S->S). “Adherent” groups were incubated two days in static monolayer culture, followed by six days of RPM exposure. Furthermore, the medium was additionally supplemented with 20 mM gadolinium or left untreated. After eight days, the samples were collected for analysis. Thereby, for the “adherent” groups, the adherent cells (A->S) were collected separately from suspended cells (A->S). In addition, cells were collected immediately after seeding which were considered to represent the “native” chondrocytes.

Visual inspection of the cells upon termination of the experiment revealed that, in static culture, chondrocytes adhered to the flask and adapted an elongated and spread-out morphology. In contrast, in RPM-exposed samples, many suspended cell clusters could be observed. These clustered cells revealed a round morphology. The RPM samples, which were allowed to attach for two days in the static condition prior to RPM exposure, also had cells that were still attached to the flask bottom. These attached cells displayed a similar morphology to the control cells in static culture.

**Figure 2:**
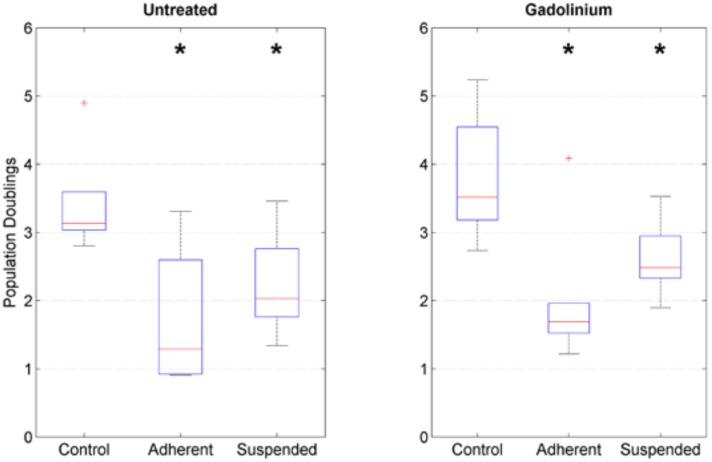
Cell proliferation. Population doublings were estimated by manual counting. RPM-exposed samples proliferated more slowly than control samples in monolayer culture. The box plot indicates the median (red central line), the 25th and 75th percentiles (box edges) and the most extreme data points (whiskers). Outliers are plotted individually. The asterisks indicate statistically significant difference as compared to the respective control group. The nomenclature of the samples is indicated in Figure 1.

**Figure 3:**
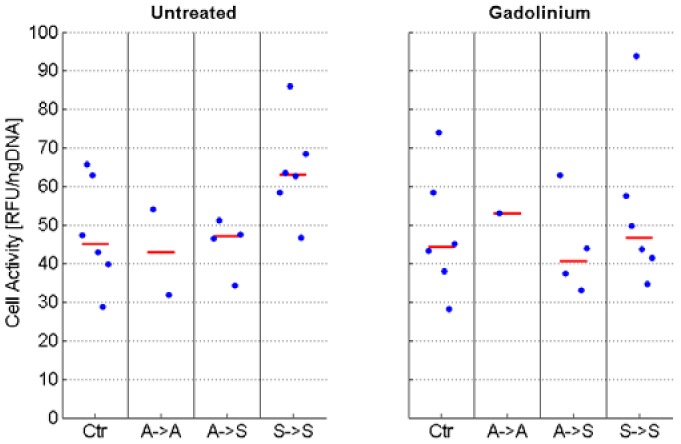
Cell activity. Chondrocytes were centrifuged to a pellet and incubated for three hours in resazurin containing culture medium, and fluorescence values were determined with a plate reader. Subsequently, the cells were digested using papain, and the DNA content was determined by Hoechst staining. Fluorescence values from the resazurin assay were normalized to the DNA content. The assay was performed in triplicates, of which the median was used for further analysis. The blue dots indicate the values from the individual experiments, whereas the red line indicates the median of all experiments. Due to the limited amount of collected cell material, the assay could not be performed for all conditions in all of the experiments. No statistically significant difference was detected among the various conditions. The nomenclature of the samples is indicated in Figure 1.

Cell proliferation was estimated by manual counting (in counting chambers). Within the eight-day culture period, the control cells in static culture underwent approximately three to four population doublings on average. In contrast, RPM-exposed samples proliferated more slowly and divided about two times on average (Figure 2). The cell activity assay using resazurin revealed no statistically significant difference among the various conditions (Figure 3). However, the assay could not always be done in all conditions due the limited amount of collected cell material.

### 2.1. Stability of Reference Genes

Five common reference genes have been used in this study: *18S*, *B2M*, *GAPDH*, *HPRT1*, and *L30*. Their stability was analyzed and ranked according to the methods of Silver et al. [57], GeNorm [58], NormFinder [59], and BestKeeper [60]. *18S*, *B2M*, and *HPRT1* were the three most stably expressed reference genes and were used in the following qPCR data analysis (Table 2). *GAPDH* was unstable and should not be used for such experiments. *18S* was also found to be stably expressed in mechanically loaded chondrocytes previously [61].

### 2.2. Phenotype

**Figure 4:**
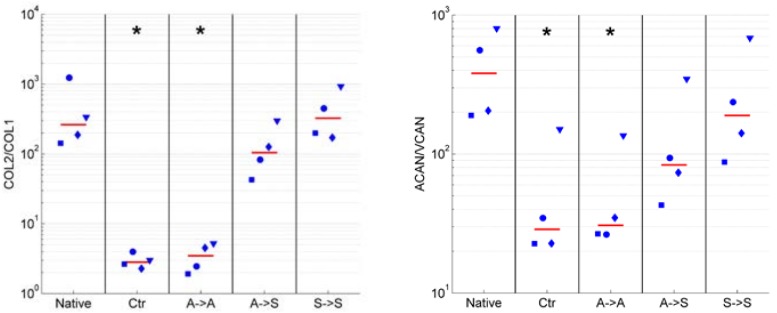
Chondrocyte dedifferentiation marker. The degree of dedifferentiation was quantified by the mRNA expression ratios of collagen type II to type I (*COL2*/*COL1*; **left**) and aggrecan to versican (*ACAN*/*VCAN*; **right**). High values of these ratios indicate differentiated chondrocytes, whereas low values indicate dedifferentiated cells. The ratios *COL2*/*COL1* and *ACAN*/*VCAN* were reduced in adherent cells (Ctr and A->A) and remained high in suspended cells (A->S and S->S). The blue markers indicate the individual values of the four unique animals (unique shape for each animal), and the red line indicates the median from all of the experiments. The asterisks indicate statistically significant difference as compared to the native cells. The nomenclature of the samples is indicated in Figure 1.

**Figure 5:**
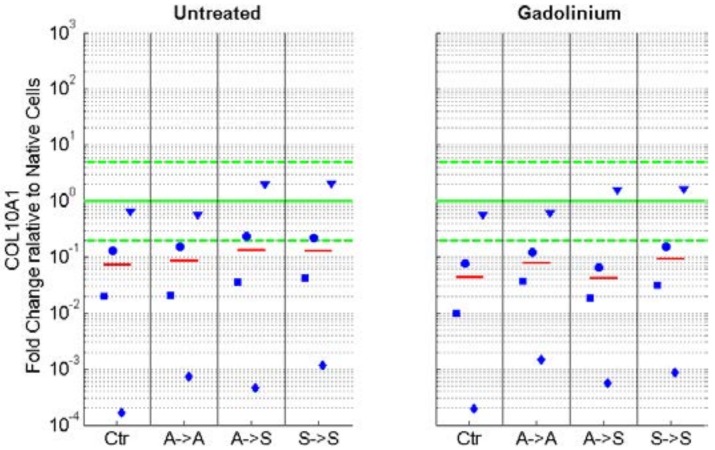
mRNA expression of collagen type X (*COL10*), normalized to the gene expression of native cells. Collagen type X expression was generally downregulated in all conditions but showed great variability among the four animals. The blue markers indicate the individual values of the four unique animals (unique shape for each animal), and the red line indicates the median from all experiments. The green horizontal lines indicate the one-fold (no change; solid line) and five-fold upregulation and downregulation (dashed lines), respectively. The nomenclature of the samples is indicated in Figure 1.

We assessed if exposure to RPM rotation preserves the phenotype in articular chondrocytes. The degree of dedifferentiation was quantified by the commonly used expression ratios of collagen type II to type I (*COL2*/*COL1*) and the proteoglycan ratios of aggrecan to versican (*ACAN*/*VCAN*). High values of these ratios indicate differentiated chondrocytes (of native healthy cartilage), whereas low values indicate dedifferentiated cells [12,16,17,21]. The ratios of *COL2*/*COL1* and *ACAN*/*VCAN* were clearly reduced in adherent cells, regardless of whether the cells were cultured in the static condition (Ctr. group) or on the RPM (“adherent” group; Figure 4). On the other hand, these ratios remained high in suspended cells (“suspended” group). Chondrocytes, which were initially seeded as adherent cells and eventually became suspended on the RPM, revealed lower *COL2*/*COL1* and *ACAN*/*VCAN* ratios than permanently suspended cells, but they clearly revealed higher ratios than adherent cells did (Figure 4). The expression of collagen type I (*COL1*), collagen type II (*COL2*), aggrecan (*ACAN*), and versican (*VCAN*) relative to the native cells is illustrated in the supplementary material (Figures S1–S4). Collagen type X (*COL10*) expression was generally downregulated in all conditions but displayed great variability among the four animals (Figure 5).

**Figure 6:**
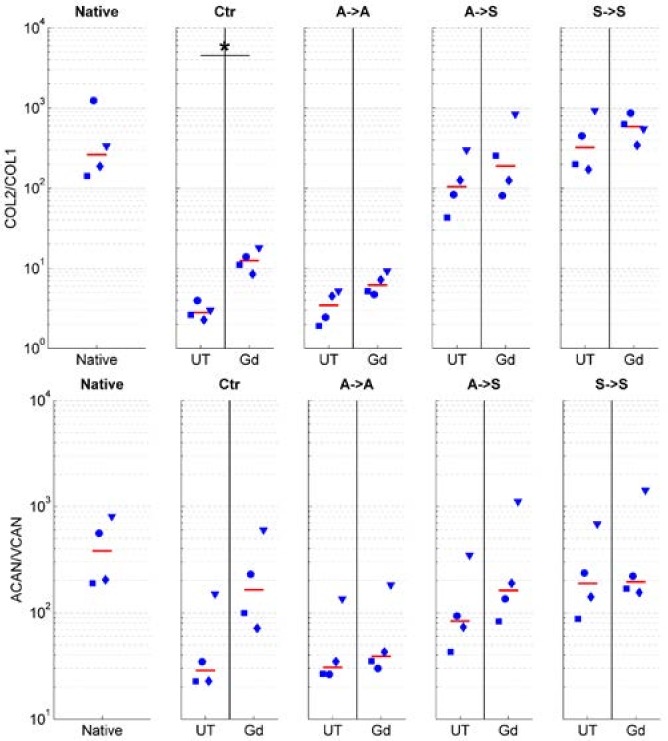
Influence of 20 mM gadolinium on chondrocyte dedifferentiation marker. The degree of dedifferentiation was quantified by the mRNA expression ratios of collagen type II to type I (*COL2*/*COL1*; **top**) and aggrecan to versican (*ACAN*/*VCAN*; **bottom**). High values of these ratios indicate differentiated chondrocytes, whereas low values indicate dedifferentiated cells. The application of gadolinium resulted in a higher *COL2*/*COL1* and *ACAN*/*VCAN* expression compared with the untreated cells. The blue markers indicate the individual values of the four unique animals (unique shape for each animal), and the red line indicates the median from all of the experiments. The asterisks indicate a statistically significant difference between the gadolinium-treated and corresponding untreated group. The nomenclature of the samples is indicated in Figure 1.

The addition of 20 mM gadolinium to the culture medium resulted in a higher *COL2*/*COL1* and *ACAN*/*VCAN* expression when compared with the untreated cells (Figure 6) but did not influence the expression of collagen type X (Figure 5).

### 2.3. Ion Channels

**Figure 7:**
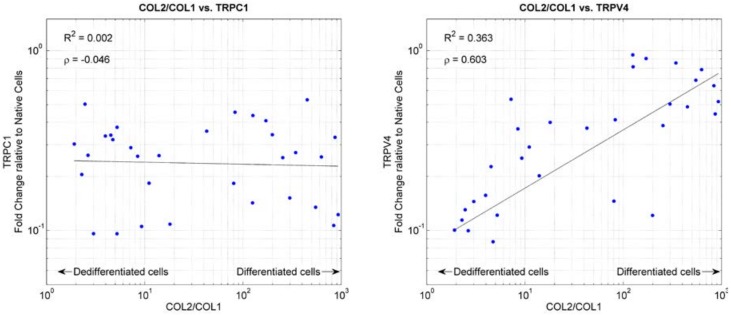
mRNA expression of *TRPC1* (**left**) and *TRPV4* (**right**) plotted against the chondrocyte dedifferentiation marker *COL2*/*COL1*. High values of *COL2*/*COL1* indicate differentiated chondrocytes, whereas low values indicate dedifferentiated cells. The mRNA expression of *TRPC1* and *TRPV4* was normalized to the respective gene expression of native cells. The black line indicates linear regression of the 10-base logarithm transformed data. *R*^2^ indicates the corresponding coefficient of determination. The *ρ*-value indicates the Pearson’s linear correlation coefficient of the 10-base logarithm transformed data.

The gene expression of the two mechanosensitive ion channels *TRPC1* and *TRPV4* was determined. Relative to native cells, *TRPV4* was downregulated in all conditions, but it was more downregulated in adherent cells than in suspended cells (Figure S5). *TRPV4* expression plotted against dedifferentiation marker *COL2*/*COL1* indicated that *TRPV4* expression roughly correlated with chondrocyte differentiation (Figure 7). *TRPV4* expression was increasingly downregulated with progressing dedifferentiation. On the other hand, *TRPC1* expression was downregulated around fivefold to 10-fold in all of the conditions and was independent of the cells’ dedifferentiation (Figure 7 and Figure S6). Overall, the application of gadolinium did not influence the expression of *TRPC1* and *TRPV4*.

## 3. Discussion

Chondrocytes formed cell clusters on the RPM, which was observed in previous studies as well [41,42]. These clusters were formed on the RPM, regardless of whether the cells were allowed to attach to the flask’s bottom for two days or were seeded directly as suspended cells. RPM rotation leads to complex and non-deterministic fluid currents in the flasks [62]. These currents probably helped to detach the cells from the flask’s bottom. Suspended cells had a higher *COL2*/*COL1* ratio than the control cells grown in monolayer culture. This finding is also in agreement with previous studies on the RPM [39,41] and the related rotating wall vessel (RWV) [45,46]. In contrast, RPM-exposed cells, which remained adherent, had a very similar phenotype to the control cells. Therefore, we conclude that RPM-exposed chondrocytes preserve their phenotype only (as assessed by the *COL2*/*COL1* and *ACAN*/*VCAN* ratios) if the cells go into suspension. The RPM rotation alone has no or only minor influence.

The clustered suspended cells displayed a round morphology, whereas control cells in the monolayer culture spread out to a fibroblast-like morphology. An early study on chondrocyte dedifferentiation using polyHEMA coated dishes already suggested that chondrocytes retain their phenotype when prevented from attaching to the substrate. Also, the cells did not acquire a spread-out morphology [63]. Similarly, chondrocytes that were cultured on SeaPlaque low-melting-temperature agarose clustered and retained their phenotype [64]. Likewise, chondrocytes cultured on collagen type I and type II were stained positive for collagen type II if the cells displayed a round morphology [65,66]. Furthermore, chondrocytes that were seeded in a three-dimensional scaffold or in pelleted culture have a round morphology and seemed to retain their phenotype [14,15,22,23,24,25,26,27,28]. However, these phenotype-preserving culture conditions also lead to reduced cell proliferation [64]. This also indicates that proliferation and maintaining the phenotype are mutually exclusive processes: this corresponds with the mutual exclusion of terminal differentiation and proliferation. These findings suggest that cell morphology could be a direct regulator of the chondrocyte phenotype. Indeed, it was already described decades ago that cell morphology could directly regulate cellular processes, including gene expression and cell proliferation. These findings lead to the formulation of the so-called tensegrity model [67,68,69,70]. In agreement with this hypothesis, chondrocytes, which were cultured for 90 days in suspension on the RWV, formed about 1 cm-wide cell aggregates [46]. Sections of these aggregates reveal a large central matrix that strongly stains for collagen type II, and in which chondrocytes display a round morphology. The outermost layer takes the form of a fibrous capsule with flattened, elongated cells and stains positive for collagen type I [46].

Because the expression of several TRP channels have shown to change with progressing dedifferentiation [51,52], we speculated that the two mechanosensitive ion channels *TRPC1* and *TRPV4* are involved in the dedifferentiation process. Therefore, the mRNA gene expression of *TRPC1* and *TRPV4* was analyzed, and the experiment was conducted under the influence of 20 mM gadolinium as well. Gadolinium is known as an unspecific ion channel blocker, also described to block mechanosensitive ion channels [71,72] and calcium-permeable channels [73,74,75,76,77,78]. The application of 20 mM gadolinium led to a slight increase in *COL2*/*COL1* and *ACAN*/*VCAN* ratios, and did not markedly influence the cell cycle in this study. How gadolinium affects the chondrocytes is unknown, but it seems to have a minor influence on the dedifferentiation process.

The function of *TRPC1* is not fully understood. It has been described to be activated by the depletion of intracellular calcium-stores (store-operated calcium influx), interactions with inositol-1,4,5-trisphosphate receptors (IP_3_Rs) and mechanical stretch (stretch activated; reviewed in [79]). In this study, the gene expression of *TRPC1* was reduced around fivefold to 10-fold in all conditions relative to native cells. This is in disagreement with a previous study on passaged chondrocytes of osteoarthritic patients, in which no difference in *TRPC1* gene expression was observed [52]. The reason for the conflicting findings is unknown, but could be explained by (1) the different species or (2) the fact that in the previous study, chondrocytes from osteoarthritic patients were used, whereas chondrocytes from young, presumably healthy cattle were used in this study.

Interestingly, we observed that *TRPV4* expressions roughly correlate with chondrocyte differentiation marker *COL2*/*COL1*. *TRPV4* is known to be activated in response to moderate heat (24–38 °C), hypotonic environments, and membrane stress (reviewed in [80]). *TRPV4* is highly expressed in chondrocytes [54,55]. In this cell type, *TRPV4* has been described to play a central role in the cellular response to osmotic challenges [81,82,83,84]. In addition, *TRPV4* was described to be involved in the metabolic response of dynamically loaded chondrocytes [85]. Our finding, which *TRPV4* expression decreases with progressing dedifferentiation, is in contradiction to a previous study on equine articular chondrocytes. Western blots revealed no difference in *TRPV4* expression over three passages in monolayer culture [51]. In support of our finding, *TRPV4* has been shown to regulate *SOX9*, a transcription factor of multiple cartilage-specific extracellular matrix molecules [56]. *TRPV4* also co-expresses with collagen type II and aggrecan during chondrogenesis [56]. At this point, we can only speculate about the role of *TRPV4* in chondrocyte dedifferentiation. However, *TRPV4* could play a key role in osteoarthritis and cartilage repair (reviewed in [86]).

## 4. Materials and Methods

### 4.1. Random Positioning Machine (RPM)

**Figure 8:**
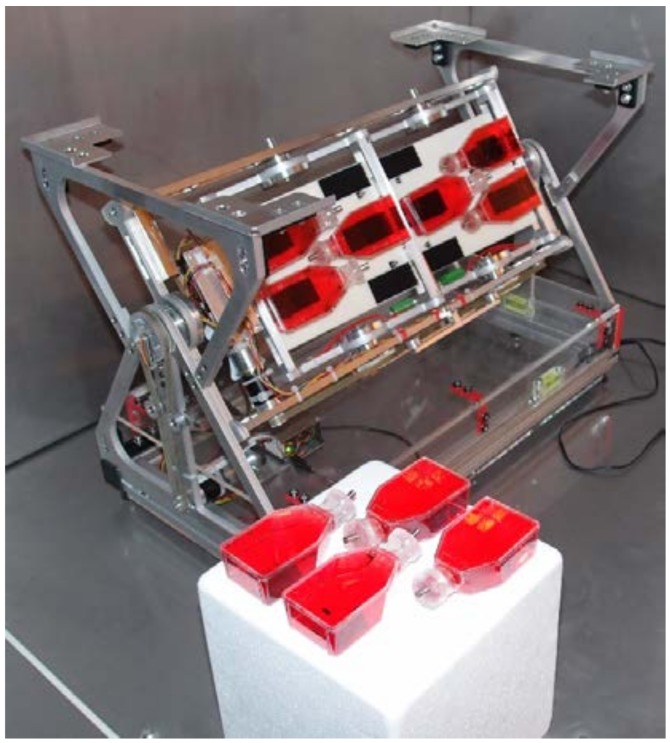
Random positioning machine (RPM). The RPM consists of a gimbal-mounted platform, which rotates samples continuously around two perpendicular axes. Samples were closed with a custom-made silicone plug and a stainless steel pin and subsequently fixed with Velcro onto the RPM. The Lucerne School of Engineering and Architecture developed the RPM.

As mentioned previously, the RPM was initially developed as a ground-based model for long-term microgravity simulation [37,47,48,49]. It consists of a gimbal-mounted platform, which allows for rotating samples continuously around two perpendicular axes (Figure 8). The two axes are each driven by electrical engines, which operate independently of each other. A custom-made software running on a laptop controls both engines [87]. In this study, the platform was rotated at a constant velocity, which was set to 60 deg/s, but the rotation direction was frequently inverted at random time points, as described previously [87]. The transition from forward to backward took place at 10 deg/s^2^ [87]. The RPM that was used in this study was developed by the Lucerne School of Engineering and Architecture (Figure 8) [37]. Samples were fixed with Velcro (Dual Lock; 3M, Saint Paul, MN, USA) onto the RPM. 

### 4.2. Bovine Chondrocytes and Cell Culture

Bovine chondrocytes were isolated from the fetlock joint of cattle [88,89] from a local abattoir. Fresh joints were opened, and the articular cartilage was scraped off from the joint using a scalpel blade. The obtained cartilage was dissected to small millimeter-size pieces and was subsequently incubated for two hours in pronase (Roche, Basel, Switzerland), with shaking taking place at 37 °C. The cartilage was then washed three times with phosphate-buffered saline (PBS) and was incubated overnight in collagenase II (Worthington, Lakewood, NJ, USA) adjusted to an activity of 600 U/mL, with shaking at 37 °C. The released cells were separated by a cell strainer from remaining tissue pieces and washed two times with PBS. Suspended cells were finally frozen and stored at −80 °C or in liquid nitrogen until further use.

The cells were cultured in a commercial T25 flask. The cell culture medium contained low glucose (1 g/L) DMEM, which was buffered with 25 mM HEPES (Gibco, Thermo Fisher Scientific, Waltham, MA, USA), and was supplemented with 10% fetal cow serum (FCS; Gibco, Thermo Fisher Scientific) and 1% Penicillin Streptomycin (Gibco, Thermo Fisher Scientific).

### 4.3. Experiment Design

Frozen bovine chondrocytes were thawed and distributed to six experimental groups, according to Table 1 and Figure 1. Control groups and “adherent” groups were left for two days in static culture with 7 mL of culture medium. After two days, the medium was aspired, and the flask was completely filled with medium, avoiding bubbles. The “adherent” groups were placed on the RPM, whereas the control groups were left in the static condition. The flasks of the “suspended” groups were completely filled immediately after seeding and were subsequently placed on the RPM. In addition, the medium was supplemented with 20 mM gadolinium (Sigma-Aldrich, St. Louis, MO, USA) or left untreated. The cells were seeded in commercial T25-flasks at a density of 125,000 or 250,000 cells per flask for the control groups or the RPM-exposed groups respectively. The flasks were closed with custom-made silicon plugs after being almost completely filled with medium. The plugs each featured a small central hole, through which the flasks were fully filled and sealed with a stainless-steel pin.

After eight days, the samples were collected for further analysis. To estimate cell proliferation, the cell concentrations were determined by manually counting cells in a counting chamber. If the harvested cell amount was sufficient, then some cells were used for a cell activity assay (see below) right after cell collection. The remaining cells were lysed for mRNA gene expression (see below). For the “adherent” groups, which were in static culture for two days and were then exposed to the RPM for six days, the adherent cells were collected separately from the cells that became suspended. In addition, freshly thawed cells were lysed for gene expression analysis immediately after seeding. These samples were considered to represent the “native” chondrocytes. Due to the large amount of medium in the flasks, a medium exchange was not necessary. The experiment was repeated four times, with cells originating from four individual animals.

### 4.4. Cell Activity Assay

The cell activity assay was performed in pellet culture because the cells that were suspended on the RPM did not adhere to the plastic of the multiwall plates, even after a few hours. Right after cell harvesting, 50,000 cells were transferred into 1.5 mL-Eppendorf tubes, topped with 0.5 mL of cell culture medium, and centrifuged at 500 g for five minutes to form a cell pellet. The cells were kept at 37 °C until all of the samples were ready. Subsequently, the tubes were centrifuged again (to stabilize the pellet), and the supernatant was removed and replaced with 500 μL of cell culture medium containing 200 μM resazurin (also known as alamar blue; Sigma-Aldrich) [90]. The samples were incubated in the dark for three hours at 37 °C and 5% CO_2_ with open lids to allow for gas exchange. After this incubation, the samples were centrifuged again (500 g for five minutes), and 400 μL of supernatant was transferred to a 48-well plate. In addition, 400 μL of fresh resazurin solution (not exposed to cells) was transferred to the well plate (blank sample). Fluorescence values were determined with a plate reader (Spark 10M, Tecan, Männedorf, Switzerland) using an excitation wavelength of 580 nm and an emission wavelength of 595 nm.

The fluorescence readings were normalized to the DNA content, as determined by Hoechst staining. The remaining medium in the sample tubes was aspired, and the pellet was washed once with 1 mL PBS (phosphate-buffered saline). Finally, the pellet was resuspended in 100 μL papain digestion solution containing 150 mM NaCl, 55 mM Na_3_-Citrate, 5 mM EDTA (ethylenediaminetetraacetic acid), 0.8 mg/mL Cystein-HCl, and papain adjusted to an activity of 3.9 U/mL. The samples were incubated at 60 °C overnight. Digested samples were transferred to a 96-well plate and were supplemented with 150 μL Hoechst solution containing 0.2 μg/mL Hoechst 33258 (bisBenzimide; Sigma-Aldrich) in PBS. After incubating for five minutes at room temperature in the dark, DNA was quantified in a plate reader (Spark 10M, Tecan) using an excitation wavelength of 360 nm and an emission wavelength 465 nm. Fluorescence values were compared with a DNA standard on the same well plate to calculate the DNA content. Fluorescence values from the resazurin assay were subtracted by the blank sample value and were subsequently divided by the DNA content. The assay was performed in triplicates, of which the median was used for further analysis.

### 4.5. mRNA Gene Expression by qPCR

RNA lysates were homogenized with the QIAshredder kit (Qiagen, Hilden, Germany), and the total RNA was extracted using the RNeasy kit (Qiagen) with DNase (Qiagen) treatment, according to the manufacturer’s instructions. cDNA was synthesized from 500 ng of total RNA using the SuperScript III reverse transcriptase (Invitrogen, Waltham, MA, USA). cDNA was diluted 20-fold and mixed with qPCR reaction solution containing SYBR Green PCR Master Mix (Applied Biosystems, Foster City, CA, USA) and 250 nM primers (Table 3). qPCR was run on an IQ5 cycler (Bio-Rad, Hercules, CA, USA), using a three-step protocol with a denaturation temperature at 95 °C (15 s), an annealing temperature at 60 °C (30 s), and an elongation temperature at 72 °C (30 s) for 40 cycles, followed by melting curve analysis. *C*_t_ values were normalized (Δ*C*_t_) to the geometric mean of the three reference genes, *18S*, *B2M*, and *HPRT1* (see also Results section). Change in gene expression was calculated using the ΔΔ*C*_t_-method [91].

### 4.6. Statistics

The data was analyzed and plotted using the software MATLAB (R2012b). For the estimated cell proliferation, cell activity, as well as the mRNA expression ratios of *COL2*/*COL1* and *ACAN*/*VCAN*, the differences between the various conditions was assessed by the nonparametric Wilcoxon rank sum test. *p*-values that were smaller than 0.05 were considered to be statistically significant.

Correlation of the relative mRNA expression changes of *TRPC1* and *TRPV4* versus the mRNA expression ratios of *COL2*/*COL1* was assessed. The linear regression of the 10-base logarithm transformed data and the corresponding coefficient of determination (*R*^2^) was computed. In addition, the Pearson’s linear correlation coefficient of the 10-base logarithm transformed data was determined.

## 5. Conclusions

In conclusion, this and previous studies revealed that the chondrocyte phenotype is preserved when the chondrocytes remain in suspension and aggregate to cell clusters. Exposure to mechanical unloading by RPM rotation alone does not preserve the chondrocyte phenotype. Results from this and previous experiments also indicate that chondrocytes retain their phenotype when retaining a round morphology. This holds true, regardless of whether the round morphology was induced by a three-dimensional scaffold [15,22,23,24,25,26,27], by pellet culture [14,25,28], by culture chamber coating [63,64,65,66], or by suspended cell culture using the RPM [39,42] or the rotating wall vessel (RWV) [45,46]. However, the culture of such cell aggregates on the RPM or the related RWV is expected to be superior as compared to standard pellet culture, due to the enhanced convection [62]. The beneficial effects of such an RPM-based tissue engineering approach require further investigation. Finally, *TRPV4* gene expression decreased with progressing dedifferentiation in this study. Because *TRPV4* also regulates the chondrogenic transcription factor *SOX9* [56], *TRPV4* could indeed play a key role in osteoarthritis and the modulation of the chondrocyte phenotype [86].

## Figures and Tables

**Table 1:** Experimental groups. Chondrocytes were distributed to six experimental groups, exposing them to either static cell culture, static culture followed by RPM exposure or RPM exposure only. The medium was additionally supplemented with 20 mM gadolinium or left untreated.

Group Name	Chemical Treatment	Mechanical Treatment
Control, UT	Untreated	8 days static culture
Adherent, UT	Untreated	2 days static culture followed by 6 days RPM exposure
Suspended, UT	Untreated	8 days RPM exposure
Control, Gd	Gadolinium	8 days static culture
Adherent, Gd	Gadolinium	2 days static culture followed by 6 days RPM exposure
Suspended, Gd	Gadolinium	8 days RPM exposure

**Table 2:** Four different methods were used to rank five common reference genes according to their stability.

Stability Rank	Silver et al.	GeNorm	NormFinder	BestKeeper
Most stable	*18S*	*18S*	*B2M*	*18S*
	*B2M*	*B2M*	*18S*	*B2M*
	*HPRT1*	*HPRT1*	*HPRT1*	*HPRT1*
	*L30*	*L30*	*L30*	*L30*
Least stable	*GAPDH*	*GAPDH*	*GAPDH*	*GAPDH*

**Table 3:** List of primer pairs. If no reference is given, the primers were designed with the Primer-BLAST tool from the National Center for Biotechnology Information (NCBI).

Gene	Primer	Sequence	Ref.
*18S*	Forward	ACG GAC AGG ATT GAC AGA TTG	[92]
	Reverse	CCA GAG TCT CGT TCG TTA TCG	
*B2M*	Forward	TGC CGA GTG AAA CAC GTT ACT	
	Reverse	GTT CAA ATC TCG ATG GTG CTG CTT	
*HPRT1*	Forward	AGA CTG CCT TCA GCC CG	
	Reverse	GGT TCA TCA TCG CTA ATC ACC AC	
*L30*	Forward	AGG AAG GCT CAA CGA GAA CA	[27]
	Reverse	CGA GGA GCA GAA ACC TTC AC	
*GAPDH*	Forward	GGC GTG AAC CAC GAG AAG TAT AA	[93]
	Reverse	CCC TCC ACG ATG CCA AAG T	
*COL1A1*	Forward	ACT GTC CTA ACG CCA AAG TCC	
	Reverse	CTC CTT TCG GTC CCT CGA C	
*COL2A1*	Forward	AAA GCC TGG AAA ATC TGG CG	
	Reverse	ACC TGG GTA ACC TCT GTG AC	
*COL10A1*	Forward	GGG AGT GCC TGG ACA CAA TG	
	Reverse	AGT TCC CAC ATC GCC TTT GG	
*ACAN*	Forward	CCT CCC CGA CTG ATG CTT CTA	
	Reverse	CAC AGC TTC TGG TCT GTT GTG G	
*VCAN*	Forward	ATA AGC CGC CTT TCA AGG ACA AGA	
	Reverse	ACT TTC TGT AGT GCA TGG GCT G	
*TRPC1*	Forward	TGT ATG ATA AAG GCT ACA CTC CCA	
	Reverse	GAT GAA CGA ATG GAA GGT GTC ATT G	
*TRPV4*	Forward	TTC CGG GAA CCG TCC A	
	Reverse	ATG TCC AGA AGC ACA GGG AT	

## Supplementary Materials

The following are available online at http://www.mdpi.com/1422-0067/19/5/1289/s1.
