# Supplementary material for: Influence of Mechanical Unloading on Articular Chondrocyte Dedifferentiation
Source: Int J Mol Sci. 2018 Apr 25;19(5):1289. doi: 10.3390/ijms19051289 (PMC5983850; doi:10.3390/ijms19051289)
Supplement: Supplementary file 1 [file ijms-19-01289-s001.pdf]

Supplementary Material to

Influence of Mechanical Unloading on Articular Chondrocyte Dedifferentiation

Simon L. Wuest, Martina Caliò, Timon Wernas, Samuel Tanner, Christina Giger-Lange, Fabienne Wyss, Fabian Ille, Benjamin Gantenbein and Marcel Egli

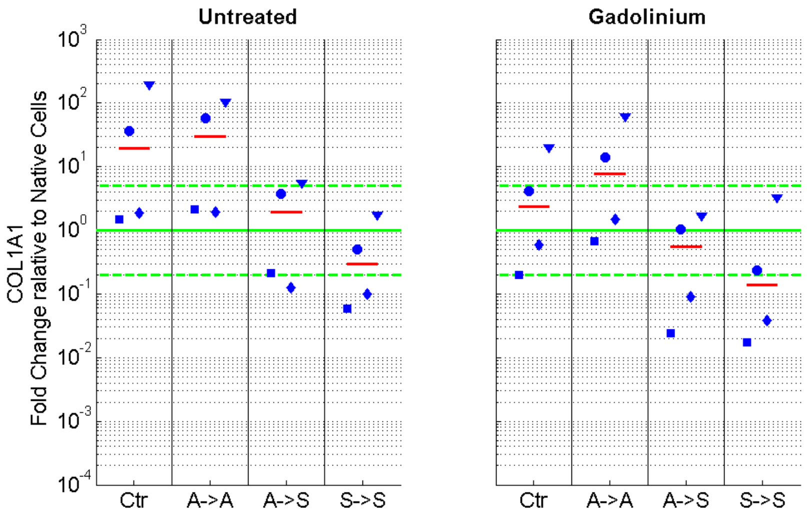

Figure S1: mRNA expression of collagen type I (*COL1*) normalized to the gene expression of native cells. The blue markers indicate the individual values of the four unique animals (unique shape for each animal), and the red line indicates the median from all experiments. The green horizontal lines indicate the one-fold (no change; solid line) and five-fold upregulation and downregulation (dashed lines) respectively. The nomenclature of the samples is indicated in Figure 1.

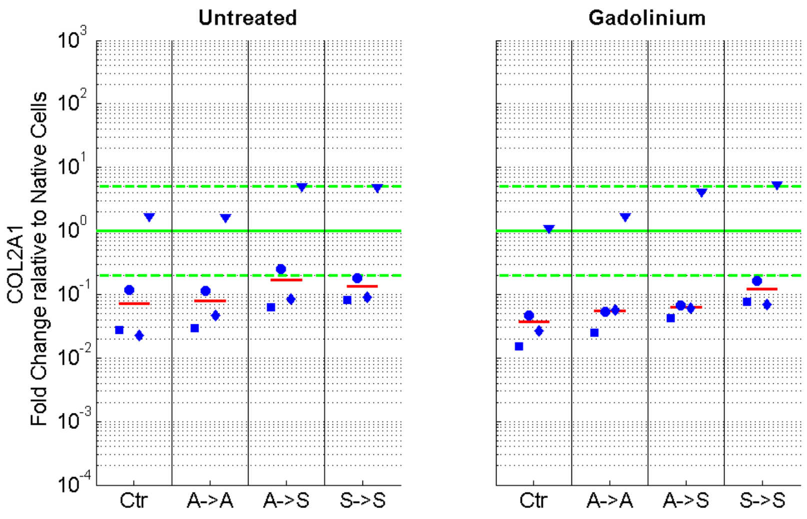

Figure S2: mRNA expression of collagen type 2 (*COL2*) normalized to the gene expression of native cells. The blue markers indicate the individual values of the four unique animals (unique shape for each animal), and the red line indicates the median from all experiments. The green horizontal lines indicate the one-fold (no change; solid line) and five-fold upregulation and downregulation (dashed lines) respectively. The nomenclature of the samples is indicated in Figure 1.

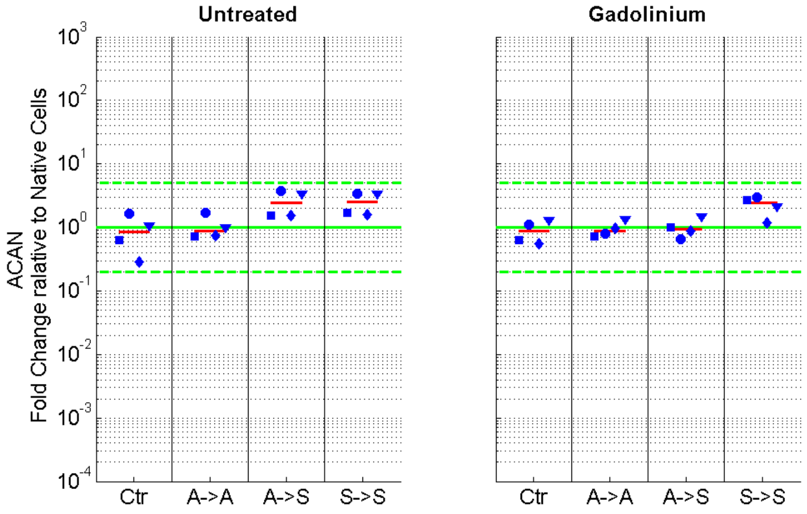

Figure S3: mRNA expression of aggrecan (*ACAN*), normalized to the gene expression of native cells. The blue markers indicate the individual values of the four unique animals (unique shape for each animal), and the red line indicates the median from all experiments. The green horizontal lines indicate the one-fold (no change; solid line) and five-fold upregulation and downregulation (dashed lines) respectively. The nomenclature of the samples is indicated in Figure 1.

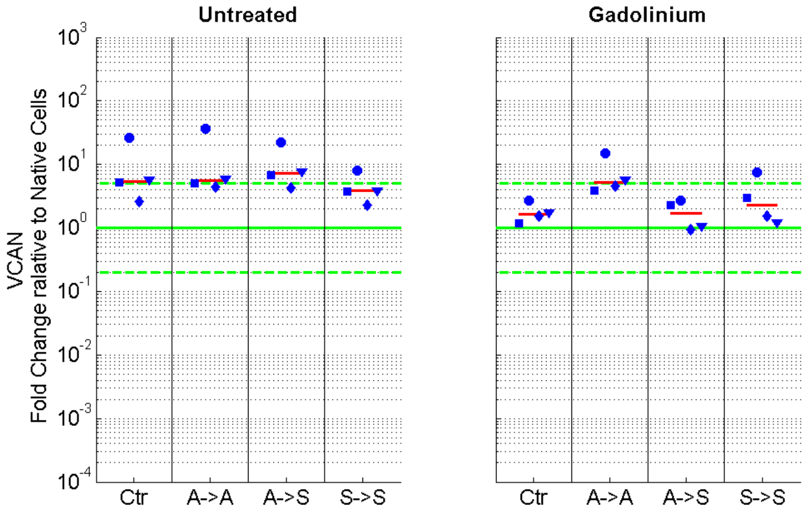

Figure S4: mRNA expression of versican (*VCAN*) normalized to the gene expression of native cells. The blue markers indicate the individual values of the four unique animals (unique shape for each animal), and the red line indicates the median from all experiments. The green horizontal lines indicate the one-fold (no change; solid line) and five-fold upregulation and downregulation (dashed lines) respectively. Nomenclature of the samples is indicated in Figure 1.

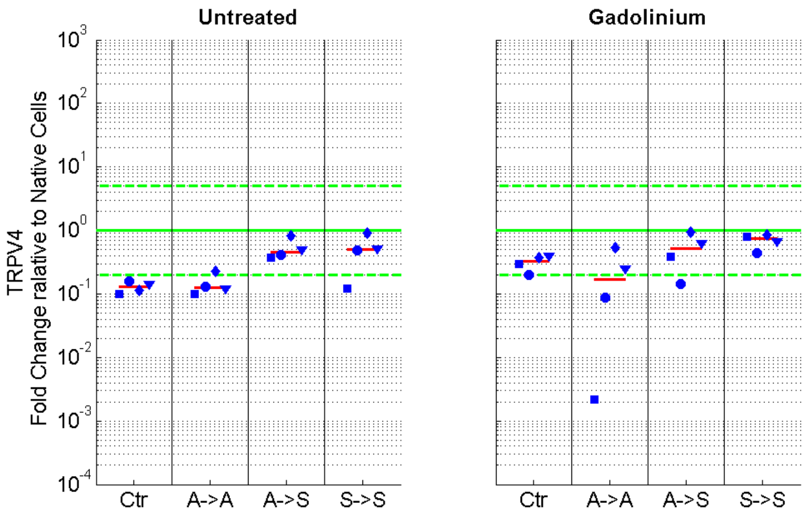

Figure S5: mRNA expression of *TRPV4* normalized to the gene expression of native cells. The blue markers indicate the individual values of the four unique animals (unique shape for each animal), and the red line indicates the median from all experiments. The green horizontal lines indicate the one-fold (no change; solid line) and five-fold upregulation and downregulation (dashed lines) respectively. The nomenclature of the samples is indicated in Figure 1.

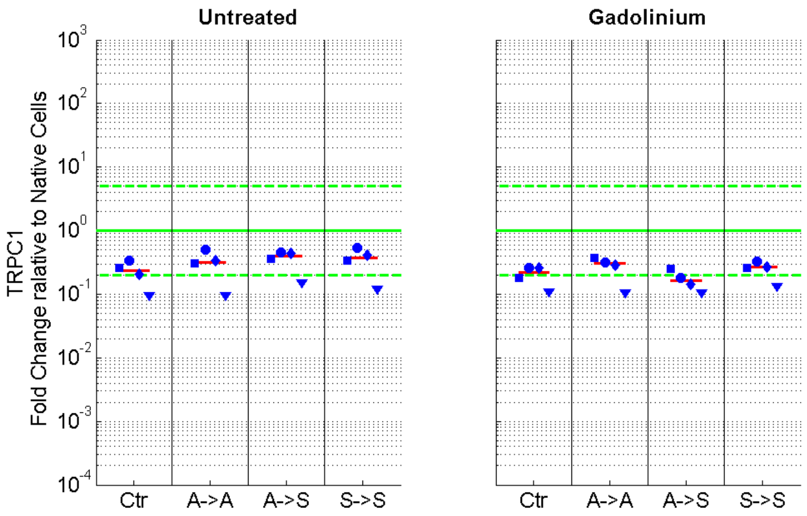

Figure S6: mRNA expression of *TRPC1* normalized to the gene expression of native cells. The blue markers indicate the individual values of the four unique animals (unique shape for each animal), and the red line indicates the median from all experiments. The green horizontal lines indicate the one-fold (no change; solid line) and five-fold upregulation and downregulation (dashed lines) respectively. The nomenclature of the samples is indicated in Figure 1.
